# Urinary Extracellular Vesicle-Derived miRNAs Reveal a Coordinated Stress-Response Network Linked to Advanced Glycation End-Products in Children and Adolescents

**DOI:** 10.3390/antiox15091150

**Published:** 2026-09-10

**Authors:** Fabio Lauria, Paola Russo, Alfonso Siani, Ivana Sirangelo, Ilenia D’Orsi, Pasquale Marena, Antje Hebestreit, Ronja Foraita, Giuseppe Iacomino

**Affiliations:** 1 Institute of Food Sciences, National Research Council, 83100 Avellino, Italy; paola.russo@isa.cnr.it (P.R.); alfonso.siani@isa.cnr.it (A.S.); ileniadorsi1990@gmail.com (I.D.); pasquale.marena@isa.cnr.it (P.M.); 2 Department of Precision Medicine, School of Medicine and Surgery, University of Campania “Luigi Vanvitelli”, 80138 Naples, Italy; ivana.sirangelo@unicampania.it; 3 Leibniz Institute for Prevention Research and Epidemiology, 28359 Bremen, Germany; hebestr@leibniz-bips.de (A.H.); foraita@leibniz-bips.de (R.F.)

**Keywords:** urinary miRNAs, extracellular vesicle (EV)-associated miRNAs, urinary AGEs, pre-clinical susceptibility signatures, obesity, children and adolescents

## Abstract

The widespread consumption of ultra-processed foods increases exposure to advanced glycation end-products (AGEs), key mediators of oxidative stress and chronic low-grade inflammation. Although AGEs contribute to adult metabolic dysfunction, their early molecular correlates in pediatric populations remain insufficiently defined. Extracellular vesicle (EV)-associated miRNAs represent stable, non-invasive indicators of systemic stress responses. This study evaluated the association between urinary AGEs and urinary EV-associated miRNAs in 94 Italian children and adolescents from the I.Family cohort. Fluorescent AGEs were quantified via spectrofluorimetry, and EV-enriched urinary miRNAs were profiled using next-generation sequencing. Differential expression and multivariable generalised linear models (adjusted for age, sex, BMI z-score, and hs-CRP) were performed. Differential expression analysis revealed a significant upregulation of hsa-miR-4516 in participants with high versus low urinary AGE levels (fold change = 2.31; FDR = 0.024). In multivariable continuous models, hsa-miR-4516 remained the primary AGE-associated miRNA, though the association attenuated following adjustment for BMI z-score and hs-CRP. Functional enrichment and network analyses highlighted pathways governing oxidative stress responses, proteostasis, and cellular survival. Overall, urinary EV-associated miRNAs may reflect coordinated biological responses to dietary AGE burden rather than serving as direct exposure biomarkers, offering integrated, non-invasive insights into early metabolic and inflammatory adaptations in children.

## 1. Introduction

Advanced glycation end-products (AGEs) comprise a heterogeneous group of bioactive compounds arising from the non-enzymatic glycation and oxidation of proteins, lipids, and nucleic acids by reducing sugars. This process, commonly referred to as the Maillard reaction or non-enzymatic browning, contributes to the formation of AGEs through two main pathways: exogenous intake and endogenous production [1]. Dietary AGEs (dAGEs) are mainly generated during high-temperature thermal food processing [2]. Upon ingestion, a fraction of dAGEs escapes degradation, enters circulation, and accumulates in tissues, contributing to the total AGE pool [3,4]. This accumulation may be particularly relevant in individuals adhering to Western dietary patterns, characterised by an overreliance on ultra-processed foods and thermally processed products [5,6].

In parallel with dietary exposure, AGEs are continuously formed endogenously under physiological conditions [7]. However, their production is markedly exacerbated by hyperglycemia, oxidative stress, and lipid peroxidation [8]. Although AGE levels are generally low in healthy young individuals, they progressively increase with ageing and under metabolic stress conditions. Accumulating evidence indicates that AGEs may contribute to the disruption of tissue architecture and cellular homeostasis, and elevated systemic AGE levels have been associated with several chronic conditions, including cardiovascular diseases [9,10], diabetic complications [11,12], hepatic and renal disorders [13], neurodegenerative diseases [14], and musculoskeletal impairment [15]. Yet, the lifelong accumulation of AGEs on long-lived proteins is considered a primary driver of age-related physiological decline and musculoskeletal degeneration [16,17].

Despite these established clinical associations, the molecular mechanisms linking AGEs to such diverse pathological outcomes remain incompletely understood. Current paradigms suggest that AGEs act as potent triggers of low-grade chronic inflammation and innate immune activation [18]. These effects are mediated either through direct protein cross-linking or via interaction with specific cell-surface receptors, most notably the receptor for advanced glycation end-products (RAGE) [19]. Activation of the AGE–RAGE axis triggers nuclear factor-kappa B (NF-κB) and NADPH oxidase signalling, sustaining oxidative stress and inflammation [20]. This interaction promotes sustained redox imbalance through increased reactive oxygen species (ROS) production, thereby linking AGE accumulation to oxidative stress–driven cellular dysfunction [21].

Because circulating and excreted AGE levels reflect the dynamic balance among dietary intake, endogenous formation, tissue accumulation, and detoxification, AGEs have been proposed as nutritional and metabolic biomarkers. However, the assessment of AGE burden in humans remains challenging due to the chemical heterogeneity of these compounds and the lack of universally accepted analytical standards. Among available approaches, spectrofluorimetric analysis provides a rapid and cost-effective approach to quantify fluorescent AGEs, making it suitable for large-scale epidemiological investigations [22].

In the context of gene regulation, microRNAs (miRNAs) have emerged as critical mediators of the cellular response to metabolic stressors, including oxidative stress responses and redox-sensitive signalling pathways. These small (~20–24 nucleotides) non-coding RNAs provide a layer of post-transcriptional control by binding to the 3′ untranslated regions (3′UTRs) of target messenger RNAs (mRNAs), leading to their degradation or translational inhibition [23]. Through this mechanism, miRNAs coordinate complex regulatory networks involved in metabolism, apoptosis, oxidative stress, and immune function. Dysregulation of miRNA expression has been recognised as a molecular feature of obesity and several non-communicable diseases [24,25]. Notably, miRNAs can also be released into extracellular fluids, either encapsulated within extracellular vesicles (EV) or associated with RNA-binding proteins, which protect them from degradation and allow their detection in biological fluids. In this form, circulating miRNAs may act as intercellular signalling molecules and stable, minimally invasive biomarkers of physiological and epigenetic status. Inflammatory miRNAs, often referred to as “immuno-miRs”, have been shown to modulate both innate and adaptive immune responses in inflammatory and metabolic contexts [26,27,28]. Consistent with this framework, our group recently reported an association between urinary AGEs and plasma miRNAs in children and adolescents, particularly among individuals with overweight or obesity, supporting the potential role of selected miRNAs as early, non-invasive biomarkers of AGE-related metabolic stress and low-grade inflammation during pediatric age [29].

Building on these previous findings, the present study aimed to investigate the relationship between urinary AGEs and EV-enriched urinary miRNAs in a subsample of the Italian children enrolled in the I.Family Study. Given their capacity to reflect environmental and metabolic exposures and to regulate inflammatory pathways, EV-associated miRNAs may represent a molecular link between AGE accumulation and disease susceptibility. Elucidating the interplay between AGEs and urinary EV-enclosed miRNA profiles may provide new insights into early molecular responses to metabolic stress and support the identification of non-invasive biomarkers for pediatric metabolic risk assessment [30].

To further explore the biological relevance of the miRNAs associated with urinary AGE levels, miRNA target genes were compiled from literature-curated, experimentally validated interactions and computational prediction databases, including TargetScan and miRDB. Targets were subsequently categorised according to the strength of experimental evidence. Finally, an Over-Representation Analysis (ORA) approach was applied to identify biological pathways and molecular processes significantly enriched among the target genes, thereby providing functional insight into the potential mechanisms linking AGE exposure to metabolic, inflammatory, and immune-related responses.

## 2. Materials and Methods

### 2.1. Participants

The research was carried out in a subset of children and adolescents enrolled in the Italian branch of the I.Family study, a European Commission–funded multicenter study designed to investigate determinants of dietary behaviour, lifestyle factors, and associated health outcomes in pediatric populations. Detailed information regarding the overall study design, objectives, and methodology of the I.Family study (ISRCTN registry number: ISRCTN62310987) has been published previously [31]. Eligibility criteria and general characteristics of the study population from which the current subsample was derived have been reported elsewhere [32,33,34,35,36].

The present investigation focused on evaluating the association between urinary AGEs and EV-enclosed urinary miRNAs in a sample of 100 apparently healthy children/adolescents. To ensure a representative and unbiased analysis, participants were randomly selected from the broader cohort in 2013/2014, conditional on the availability of a complete dataset comprising anthropometric measurements, urinary AGEs and high-sensitivity C-reactive protein (hs-CRP) levels. Furthermore, the randomisation process was strictly stratified to maintain a balanced distribution across sex, weight status categories defined according to the IOTF cut-off values [37], and hs-CRP levels. Ethical approval for the study was granted by the relevant national ethics committees through the local Health Authority (ASL Avellino). All study procedures were conducted in accordance with the principles outlined in the Declaration of Helsinki.

### 2.2. Anthropometric Measurements

A detailed description of the anthropometric measurements in the I.Family study, including intra- and inter-observer reliability, has been published elsewhere [38]. Briefly, weight was measured to the nearest 0.1 kg (Tanita BC420 SMA, Tanita Europe GmbH, Sindelfingen, Germany) with participants in fasting status, without shoes and with light clothing. Height was measured with a calibrated stadiometer (Seca 225, Seca GmbH & Co., KG., Hamburg, Germany) and recorded to the nearest 0.1 cm. Body mass index (BMI) was calculated by dividing body weight (in kg) by height squared (in m^2^). Age- and sex-specific BMI z-scores were calculated according to Cole and Lobstein [37].

### 2.3. Biochemical Analysis

Blood collection and biochemical analyses were performed following standardised procedures. Fasting venous blood samples were collected using BD Vacutainer^®^ tubes (Becton, Dickinson and Company, Franklin Lakes, NJ, USA). Blood matrices were centrifuged and processed within two hours of venipuncture, and the resulting serum aliquots were immediately cryopreserved at −80 °C. Details regarding sample handling, processing, and biochemical determinations have been described previously [39]. All subsequent biochemical analyses were conducted at a central laboratory facility to ensure analytical standardisation. Serum hs-CRP was measured using electrochemiluminescent multiplex ELISA assays (Meso Scale Discovery, Rockville, MD, USA).

### 2.4. Detection of Urinary Fluorescent AGEs

Morning urine samples were collected by children and adolescents at home and delivered to the study centre on the same day. When the time between collection and delivery exceeded two hours, parents were instructed to store samples at 4 °C until transport. Upon arrival, urine samples were immediately aliquoted and stored at −80 °C until analysis. AGE-associated fluorescence was quantified using a PerkinElmer Life Sciences LS 55 spectrofluorimeter (PerkinElmer, Waltham, MA, USA). Prior to analysis, urine samples were diluted 1:10 in PBS [40]. Fluorescence emission spectra were recorded between 400 and 600 nm following excitation at 370 nm at room temperature. Fluorescence intensity was corrected for background signal and quantified at the emission maximum centred at 440 nm. Results were expressed as relative fluorescence units (arbitrary units, AU) and normalised to urinary creatinine concentration (g/L) to account for variations in urine dilution. Urinary creatinine was measured by the Jaffe reaction using a COBAS INTEGRA 400 plus analyser (Roche Diagnostics Ltd., Rotkreuz, Switzerland).

### 2.5. miRNA Profiling from Extracellular Vesicle-Enclosed Urinary Samples

EV–derived miRNAs, along with other small RNA species, were isolated and profiled by high-throughput next-generation sequencing (NGS). Total RNA extraction was performed using the exoRNeasy Midi Kit (Qiagen, Hilden, Germany), following the manufacturer’s instructions. To support normalisation and monitor technical variability, 1 μL of QIAseq miRNA Library QC Spike-Ins (Qiagen) was added before the RNA extraction step. Illumina sequencing libraries were generated from 5 μL of extracted RNA using the QIAseq miRNA Library Prep Kit (Qiagen) specifically optimised for low-input biofluid-derived RNA. Library indexing was performed using the QIAseq Unique Dual Indexes Plates Set A and B (Qiagen). Library quality and fragment size distribution were assessed using an Agilent 4150 TapeStation (Agilent Technologies, Santa Clara, CA, USA) with D1000 High Sensitivity ScreenTape, while library concentrations were quantified by real-time PCR using the KAPA Library Quantification Kit (Kapa Biosystems, Wilmington, MA, USA). Sequencing was performed on the Illumina NovaSeqX system platform in 150 bp paired-end mode (Illumina Inc., San Diego, CA, USA), according to the manufacturer’s protocol. Confirmatory quantitative PCR (RT-qPCR) on selected miRNAs was performed as previously reported [29].

### 2.6. Data Processing and Statistical Analysis

Bioinformatic processing was first performed using the CLC Genomics Workbench miRNA pipeline (QIAGEN, Aarhus, Denmark). The analytical workflow included read quality filtering (removal of low-quality reads), adapter and length trimming, UMI-based read mapping, normalisation using 52 technical spike-in controls, miRNA quantification, and alignment against miRBase v22 and the human genome assembly GRCh38.p13. UMI-derived raw count matrices were generated for downstream statistical analyses.

For exploratory differential expression analysis, miRNA expression data were normalised in CLC Genomics Workbench using the Trimmed Mean of M-values (TMM) method and reported as counts per million (CPM). The initial dataset underwent stringent quality control. Principal Component Analysis (PCA) was employed to assess transcriptional variance across samples. To minimise biological noise and optimise the signal-to-noise ratio, extreme outliers (*n* = 6) were identified and excluded based on a threshold of 3 standard deviations from the mean in the first two principal components. This step was critical, as retaining such highly divergent profiles would artificially inflate the negative binomial dispersion estimates within the DESeq2 framework, thereby compromising statistical power and masking genuine biological variance.

As an initial screening step, participants were categorised into low- and high-AGE exposure groups according to age- and sex-specific median AGE values within the study population in order to preserve adequate sample size and statistical power (n = 47 per group). Differentially expressed miRNAs were identified using a Benjamini–Hochberg-adjusted *p*-value (FDR) threshold of <0.05 and an absolute log2 fold change (FC) ≥ 1. Results were visualised using hierarchical clustering and volcano plots.

To further investigate AGE-miRNA associations while preserving the continuous nature of AGE measurements and avoiding information loss due to categorisation, multivariable analyses were performed using the DESeq2 package, version 1.50.2 [41]. DESeq2 analyses were conducted on the raw UMI count matrix exported from CLC Genomics Workbench prior to TMM normalisation. Data normalisation, dispersion estimation, and differential expression testing were performed according to the standard DESeq2 workflow based on negative binomial generalised linear models appropriate for RNA sequencing count data.

The natural logarithm of AGE levels (ln_AGE) and clinical covariates (sex, age, BMI z-score, and hs-CRP) were standardised as Z-scores before model fitting to facilitate comparison of effect estimates. Three progressively adjusted models were evaluated: (1) an unadjusted model including ln_AGE only to explore overall AGE-miRNA associations; (2) a partially adjusted model including sex, age, and BMI z-score to isolate transcriptional variability associated with AGE levels independently of growth-related factors; and (3) a fully adjusted model including hs-CRP to account for systemic inflammation, allowing identification of the independent glycation-related transcriptomic component. This hierarchical modelling strategy enabled assessment of the robustness of AGE-miRNA associations across increasing levels of covariate adjustment.

**Figure 1:**
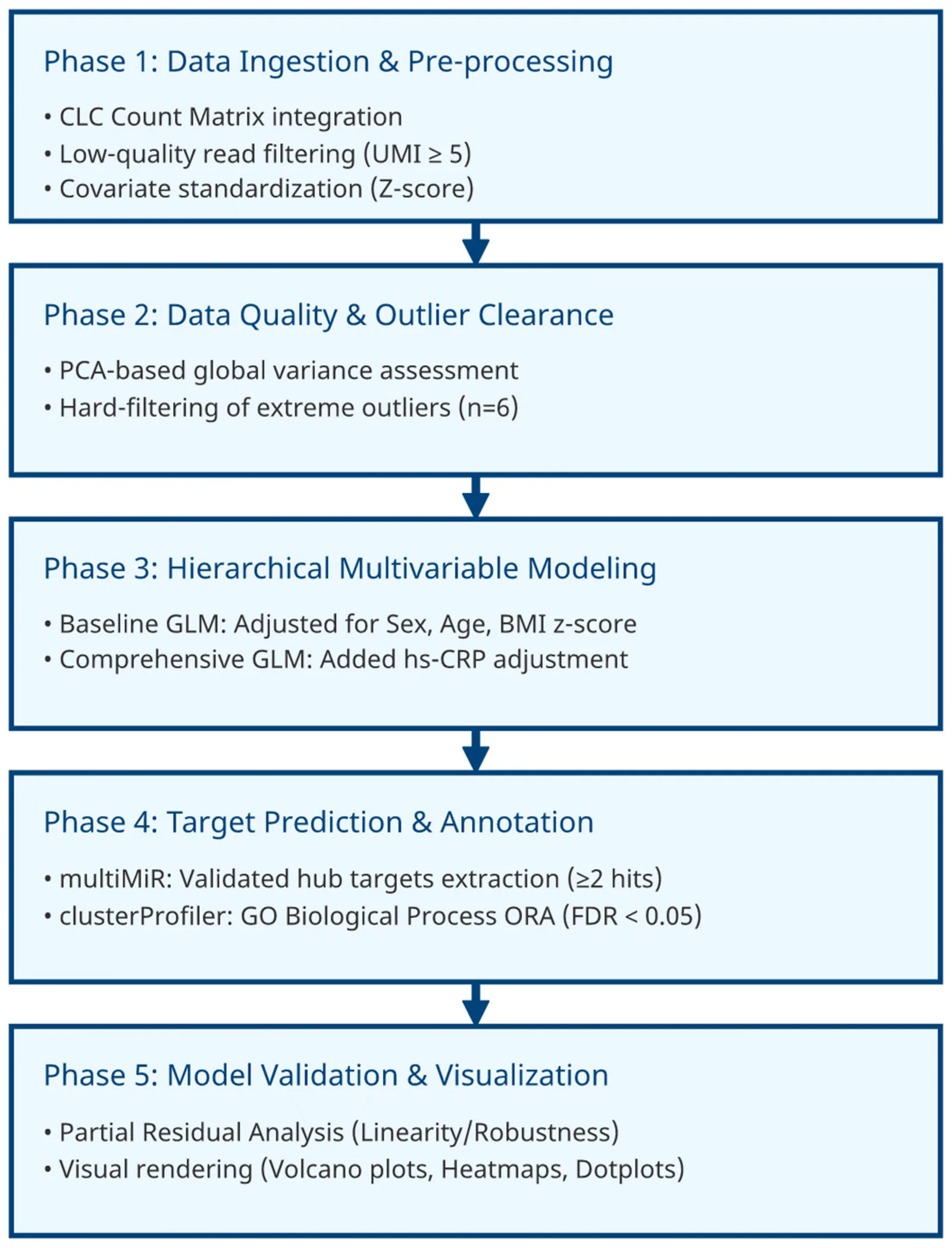
Comprehensive analytical workflow for EV-enriched urinary miRNA profiling. The flowchart illustrates the sequential bioinformatics pipeline applied to identify AGE-associated miRNA signatures. The framework progresses from raw data ingestion and stringent quality control (outlier clearance) to a hierarchical multivariable modelling approach utilising DESeq2. Downstream processing includes multiMiR-based target prediction, functional annotation (Over-Representation Analysis), and statistical validation via partial residual analysis.

To further assess model robustness, partial residual analysis was performed. This approach enabled visualisation of the relationship between the primary predictor (ln_AGE) and miRNA expression while isolating the independent effect of AGE levels after adjustment for BMI z-score, demographic (sex and age), and inflammatory (hs-CRP) covariates. This integrative framework allowed evaluation of both categorical and continuous AGE-miRNA associations (Figure 1).

miRNA tissue-specific expression was also investigated using the miRNATissueAtlas platform (Saarland University) [42].

### 2.7. Functional Annotation

miRNA target genes were compiled from literature-curated, experimentally validated interactions and computational prediction databases (TargetScan, miRDB). Targets were categorised according to the level of experimental support. To investigate the biological relevance of the identified miRNA signatures, we applied an ORA framework [43]. Since individual miRNA associations did not maintain formal FDR significance in the fully adjusted multivariable models incorporating systemic inflammation, this downstream functional analysis was conducted as an exploratory hypothesis-generating step. Specifically, target prediction was performed on the top-ranked candidate miRNAs exhibiting the lowest nominal *p*-values in the fully adjusted model. First, experimentally validated gene targets for the significant miRNAs were retrieved using the multiMiR R package [44]. To enhance the reliability of functional target identification and minimise false-positive findings, targets based solely on in silico predictions were excluded. The analysis was restricted to hub targets with robust experimental validation, including evidence from luciferase reporter assays, Western blotting, and other validated experimental approaches. Target information was collected from miRTarBase, TarBase, and miRecords, and only interactions supported by at least two independent databases were retained for subsequent analyses. Subsequently, target gene symbols were converted to Entrez IDs via the org.Hs.eg.db annotation package, version 3.22.0. Functional enrichment against the Gene Ontology (GO) Biological Process database was executed utilising the clusterProfiler R package [45]. Statistical significance for pathway enrichment was determined using a Benjamini–Hochberg-adjusted *p*-value (FDR) threshold of <0.05. Finally, the resulting enriched biological pathways were graphically rendered to visualise the respective gene ratios and enrichment significance.

## 3. Results

Of the initial 100 participants, six extreme outliers were identified and excluded from the analysis. The anthropometric and biochemical characteristics of the 94 study participants are summarised in Table 1. Males showed a significantly higher BMI z-score compared to females (*p* < 0.001), while no meaningful sex differences were observed for age, hs-CRP, or AGE levels.

**Figure 2:**
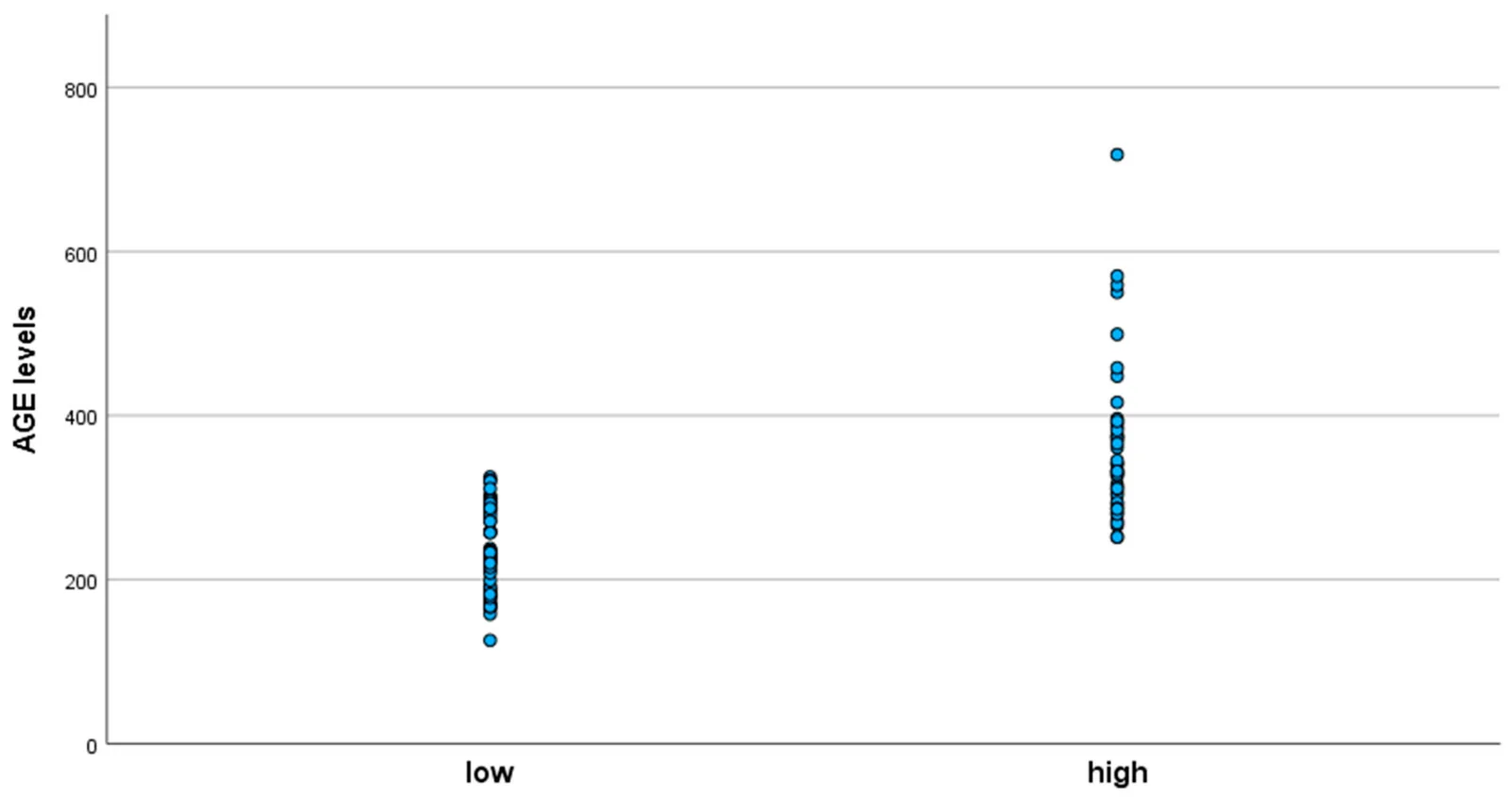
The study population was stratified into low and high AGE groups based on the median value.

Urinary AGE levels were dichotomised based on the median value of the study population, defining low and high exposure groups (Figure 2). This binarisation approach was adopted to enhance statistical robustness and to maximise contrast between individuals with lower AGE exposure and those with a higher AGE burden. This categorisation was used as the primary grouping variable for the initial differential expression screening, whereas continuous exposure data were employed for all subsequent generalised linear models.

Urinary extracellular vesicle-associated miRNAs were isolated and profiled using an untargeted high-throughput small RNA-seq procedure. All sequencing libraries met Illumina quality control specifications and were pooled at equimolar concentrations before sequencing. The mean sequencing depth was 9.47 million reads per sample, ensuring robust coverage for downstream analyses.

### 3.1. EV–miRNAs Differential Expression and Target Analysis

Primary differential expression analysis (*unadjusted approach*) between high and low AGE groups, performed using CLC Genomics Workbench, identified several miRNAs with nominal statistical significance.

**Figure 3:**
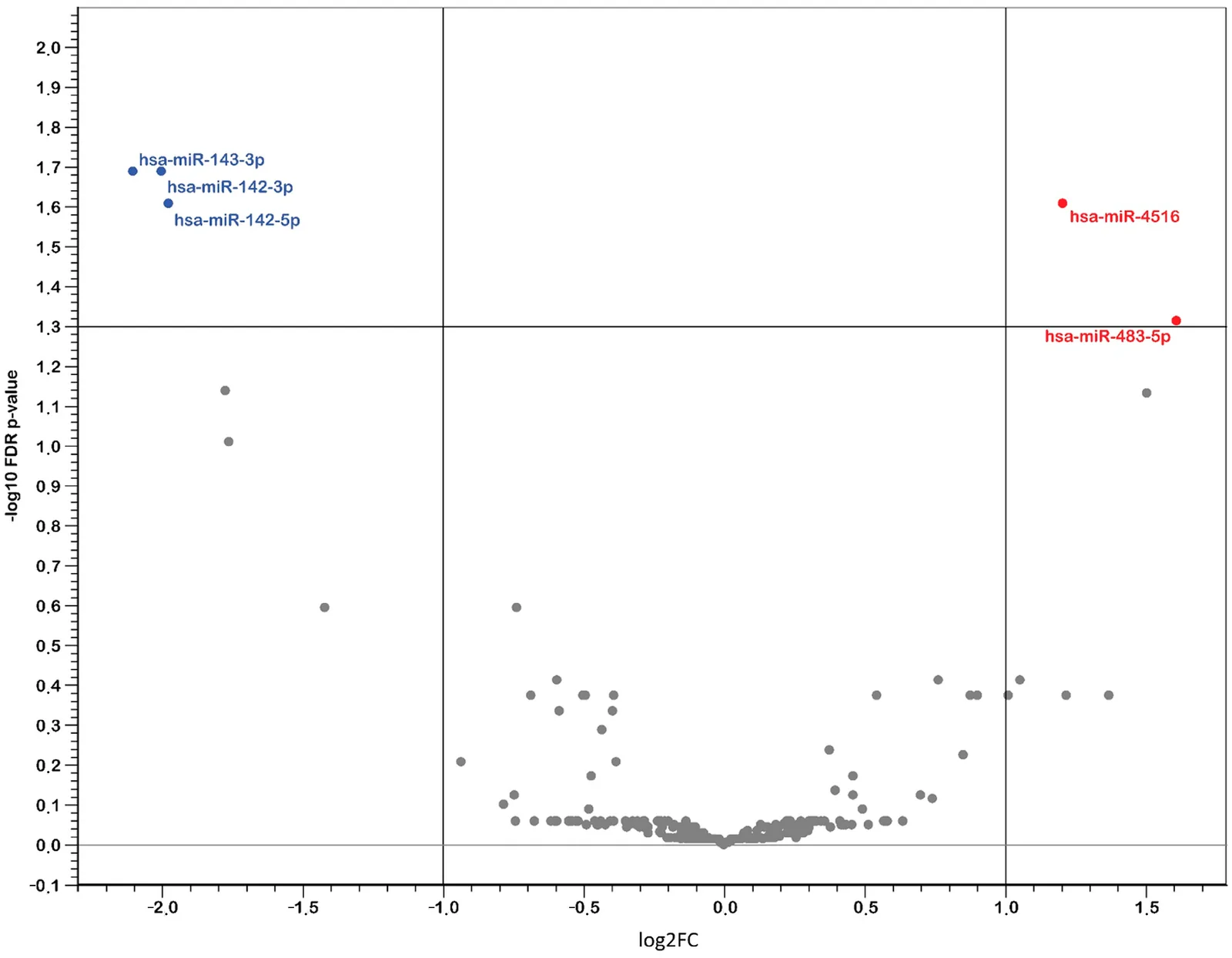
Volcano plot of differentially expressed miRNAs. The volcano plot displays the differential expression of miRNAs between the study groups. The x-axis represents the log2 fold change in miRNA expression, while the y-axis shows the −log10 adjusted *p*-value. Each dot represents an individual miRNA. Grey dots indicate non-significant miRNAs (cut-off criteria: |fold change| < 2 and FDR ≥ 0.05).

After multiple-testing correction, five putative EV-associated miRNAs remained significantly dysregulated in the high-AGE group compared with the low-AGE group. Specifically, hsa-miR-143-3p, hsa-miR-142-3p, and hsa-miR-142-5p were downregulated, whereas hsa-miR-4516 and hsa-miR-483-5p were upregulated (Table 2; Figure 3).

Overall, this pattern is consistent with an AGE-associated extracellular vesicle signature linked to immune-inflammatory dysregulation, tissue remodeling/fibrotic responses, and pathways previously associated with oxidative and mitochondrial stress, and metabolic adaptation. In particular, the reduced expression of miR-142-3p/5p and miR-143-3p may reflect disruption of regulatory programs involved in immune signalling and remodeling, whereas the increased levels of miR-4516 and miR-483-5p may reflect adaptive molecular signatures previously associated with redox and metabolic stress (Table 3).

Collectively, the FDR-significant EV-associated miRNAs identified in the high-AGE group point to convergent biological pathways involving immune-inflammatory regulation, tissue remodelling, and processes previously linked to oxidative stress, mitochondrial function, and metabolic adaptation, supporting the biological plausibility of an AGE-associated extracellular vesicle signature.

### 3.2. Impact of Covariates on Global Transcriptional Analysis by GLMs

Univariate analysis further confirmed miR-4516 as the most upregulated miRNA (FDR *p*-value = 0.046; log2FC = 0.62), in agreement with both analytical approaches, highlighting that this miRNA was consistently identified by the two bioinformatic methods (Table 4). Of note, RT-qPCR on hsa-miR-4516 also confirmed NGS results (Supplementary Figure S1).

To further distinguish between direct glycation-induced transcriptional changes and systemic inflammatory responses, a two-stage multivariable approach was adopted. The baseline model (Model 1, Supplementary Table S1) identified transcripts associated with AGE burden regardless of inflammatory status. By contrast, the comprehensive model (Model 2, Supplementary Table S2), which integrates hs-CRP, allowed the isolation of the independent transcriptional signature of AGEs.

The retention of nominal significance of miR-4516 in Model 2 is consistent with a possible role of this miRNA as a marker of AGE-induced stress, independently of acute systemic inflammatory fluctuations.

**Figure 4:**
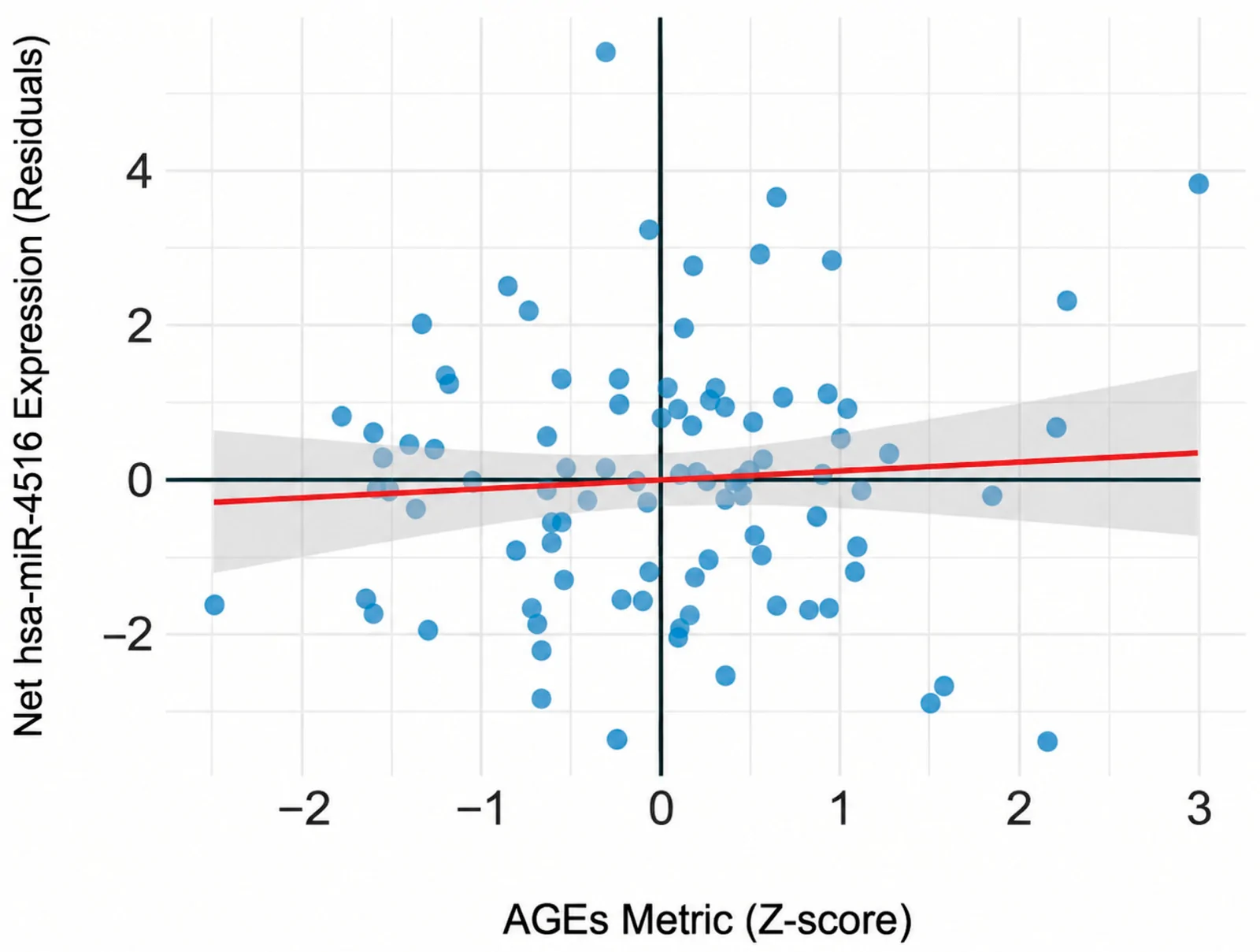
Partial residual analysis of the association between ln AGE and hsa-miR-4516 expression. The plot illustrates the net association between AGE levels (Z-score) and hsa-miR-4516 expression (residuals) within the fully adjusted comprehensive model. The expression values have been purified from the confounding effects of demographic (sex, age), anthropometric (BMI z-score), and inflammatory (hs-CRP) covariates. The red line represents the linear regression fit with the corresponding 95% confidence interval (shaded area), consistent with an independent positive association between glycation burden and miR-4516 expression.

The inclusion of these variables substantially altered the differential expression landscape: most miRNAs that were nominally associated with AGE levels in the unadjusted analysis lost statistical significance after adjustment, suggesting that miRNA variability is largely driven by hs-CRP z-scores and other systemic biological processes rather than direct AGE exposure per se. This supports a model in which AGEs may act as upstream modulators of systemic inflammatory pathways and other stress-response mechanisms, rather than directly determining miRNA expression patterns. Although no miRNA reached FDR-adjusted significance in these covariate-adjusted models, likely due to high inter-individual variability, additional biologically relevant candidates emerged based on nominal *p*-values (*p* < 0.01). In particular, hsa-let-7g-5p and hsa-miR-100-5p were identified as the most significantly downregulated transcripts with increasing AGE levels (*p* = 0.0014 and *p* = 0.0020, respectively). Of note, partial residual analysis suggested that hsa-miR-4516 variability was more closely related to inflammatory status, as reflected by hs-CRP, than to AGE levels per se. The slope observed in the partial residual plot indicates that, after controlling for inflammation and other covariates, the independent association between miR-4516 and AGE levels is preserved, although substantially attenuated (Figure 4).

Across analytical approaches, miR-4516 emerged as the most robust and consistently upregulated miRNA, representing the primary AGE-associated signal. miR-206 displayed a concordant upregulation trend but with weaker statistical support after adjustment. In contrast, other candidates, including miR-122-5p, miR-429, and miR-126-3p, showed only partial consistency, suggesting a more heterogeneous and context-dependent contribution.

### 3.3. Functional Enrichment

Functional enrichment analysis was performed on predicted and experimentally validated targets of hsa-miR-4516 to characterise the biological processes associated with EV-enriched urinary miRNAs in the context of AGEs. Gene Ontology analysis revealed a significant enrichment of pathways related to cellular stress response, apoptosis regulation, and cell proliferation. In particular, processes previously associated with oxidative stress responses, protein phosphorylation, and intracellular signal transduction were prominently represented.

Target analysis further indicated that miR-4516 may regulate key molecular networks, including PTPN14, CDKN1A, PTEN-related signalling components, and MAPK10, supporting potential roles in stress adaptation, proliferation, and cell survival. Consistently, KEGG pathway analysis highlighted enrichment in major signalling cascades such as PI3K–AKT, MAPK, and Hippo pathways (Supplementary Table S3). Overall, these findings suggest that miR-4516-associated gene networks may be involved in cellular programs related to stress response and survival signalling.

Putative tissue-specific expression was also evaluated by miRNATissueAtlas tools (Supplementary Figure S3) [42].

**Figure 5:**
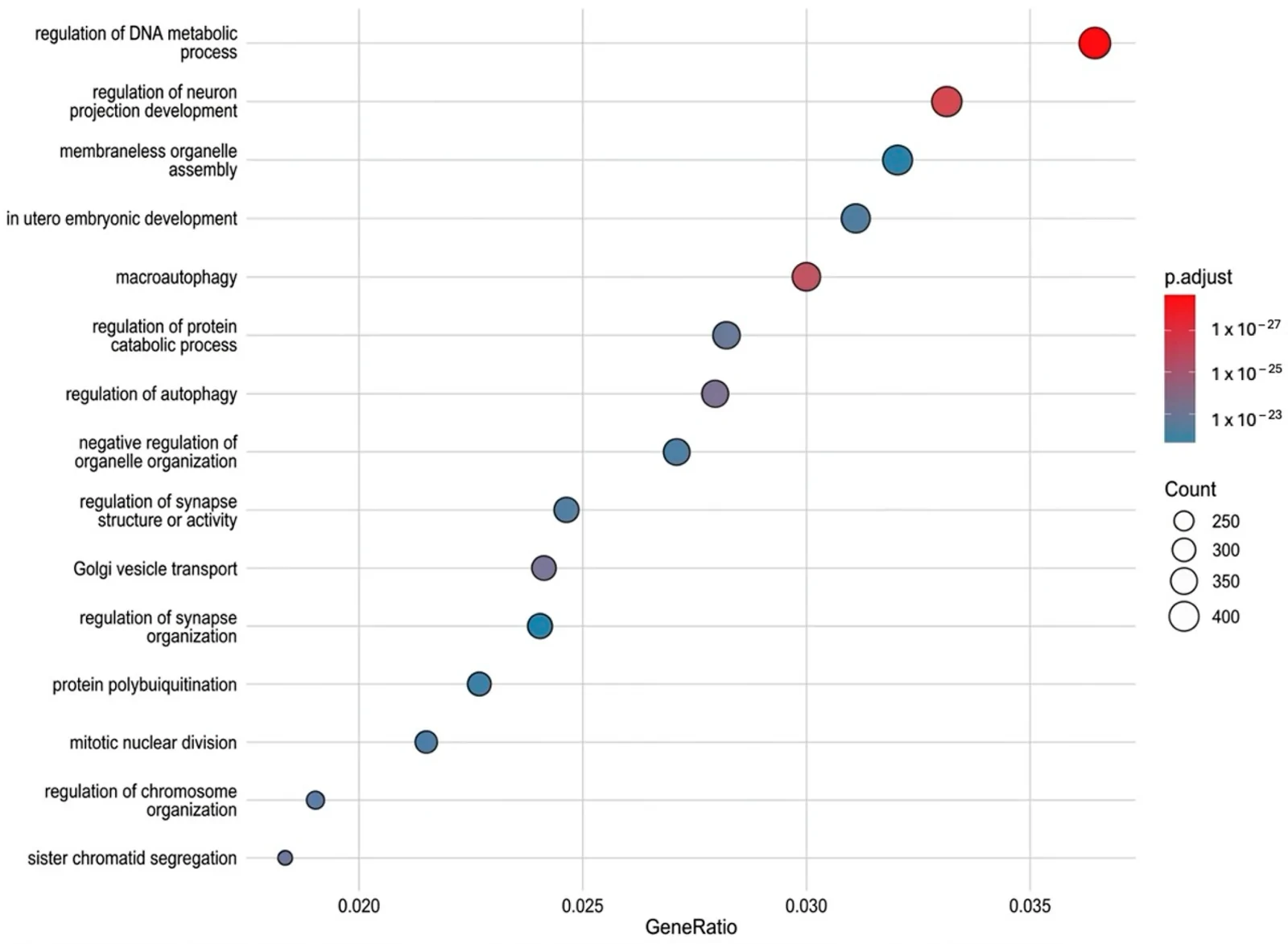
Exploratory network-level functional enrichment analysis of AGE-associated EV-miRNAs. Validated targets were strictly defined using the multiMiR package as transcripts with strong experimental evidence confirmed in at least two independent databases, excluding computational predictions. The analysis revealed significant enrichment in biological processes related to proteostasis, including macroautophagy and protein polyubiquitination, as well as cellular senescence–associated pathways. Additional enrichment in Golgi vesicle transport suggests a potential link between AGE accumulation and modulation of vesicular trafficking. Overall, the network highlights a coordinated EV-miRNA–mediated response reflecting proteotoxic and metabolic stress.

Given the attenuation of single-miRNA significance after covariate adjustment, the collective biological impact of AGE-associated miRNAs was further investigated using an exploratory approach. ORA based on validated targets shared among the highest-ranked candidates from the fully adjusted GLM (selected according to their lowest nominal *p*-values, including hsa-let-7g-5p, hsa-miR-100-5p, and hsa-miR-4516) revealed a concerted network-level regulatory response. Enriched pathways converged on proteostasis-related processes, particularly macroautophagy and protein polyubiquitination, as well as cellular senescence programs involving DNA metabolic regulation. Enrichment in Golgi vesicle transport further suggested a potential link between AGE accumulation and modulation of vesicular trafficking. Overall, these exploratory findings indicate that continuous AGE exposure may be associated with a coordinated EV-miRNA network response potentially linked to managing proteotoxic stress and facilitating the clearance of glycated proteins, rather than eliciting large independent transcriptomic changes (Figure 5). This network-level signature may represent a key biological feature of the AGE-associated EV-miRNA response. However, further functional studies are strictly required to determine whether these pathways represent direct mechanistic consequences of AGE exposure.

## 4. Discussion

The progressive adoption of Westernised dietary patterns has led to increased exposure to AGEs, which are recognised contributors to oxidative stress and chronic low-grade inflammation [5,6,18]. Although their effects have been extensively investigated in adults, their role in shaping early molecular responses during childhood remains less clearly defined. In this context, EV–associated miRNAs represent a promising class of biomarkers, as they reflect integrated biological responses to metabolic and environmental stressors [26,27,28].

In the present study, we investigated the relationship between urinary AGEs and urinary EV-associated miRNAs in a pediatric population. Among the identified candidates, hsa-miR-4516 showed the strongest initial association with AGE levels and emerged as the most consistent signal across analytical approaches. This relationship was fully attenuated after adjustment for inflammatory and anthropometric covariates, underscoring its strong dependence on systemic inflammation. Nevertheless, hsa-miR-4516 remained linked to AGE levels throughout the multivariable analyses, suggesting a complex underlying interplay that warrants further investigation. Rather than a direct exposure marker, its modulation may reflect integrated inflammatory and cellular stress-response programs associated with AGE accumulation. This interpretation is consistent with the reported involvement of miR-4516 in pathways related to cellular stress response, mitochondrial function, and inflammatory signalling [50,51,55]. In addition, regulatory mechanisms linking cellular stress to proliferation control and cell cycle progression have been described in different experimental models, highlighting the role of stress-responsive molecular networks in modulating adaptive cellular responses [56,57]. The EV-mediated transport of miRNAs supports their role in intercellular communication and systemic adaptation to stress conditions. In this context, the urinary EV compartment likely reflects a composite signal shaped by renal processing, vesicle release, and systemic metabolic status [55,58], rather than a direct readout of dietary AGE exposure.

A key finding of this study is that the association between AGEs and miRNA expression was substantially attenuated after adjustment for inflammatory and anthropometric covariates. This attenuation reveals that EV-miRNAs may primarily reflect downstream systemic responses rather than direct AGE exposure. Collectively, these findings support a model in which AGEs may act as upstream modulators of inflammatory pathways and broader cellular stress-response mechanisms that subsequently influence EV-miRNA profiles. The weakening of AGE-miRNA associations following adjustment may, at least in part, be attributable to sex-related differences in BMI and hs-CRP levels. As sex, adiposity, and inflammation are closely interconnected, the potential impact of residual confounding and reduced statistical power should also be considered. Overall, these results suggest that EV-miRNA variability is predominantly driven by systemic biological processes, particularly inflammation and pathways previously associated with cellular stress responses, rather than by glycation alone [18,47]. Importantly, the exploratory network-level analysis further supports this interpretation. The coordinated enrichment of pathways related to proteostasis, including macroautophagy and protein polyubiquitination, alongside cellular senescence and vesicular trafficking processes, indicates that AGE exposure may be associated with a structured adaptive response. These pathways are critically involved in the management of proteotoxic stress and the clearance of damaged or glycated proteins. The enrichment in Golgi vesicle transport further suggests a potential feedback mechanism linking AGE accumulation to the modulation of EV biogenesis and trafficking. From a mechanistic perspective, the convergence on proteostasis and autophagy-related pathways is particularly relevant in the context of AGE accumulation. AGEs are known to promote proteotoxic stress and protein modification, which may overwhelm cellular quality control systems. The coordinated modulation of EV-miRNAs targeting these pathways could represent an adaptive response maintaining cellular homeostasis under chronic stress.

An additional aspect relevant to interpretation is the atypical biogenesis of hsa-miR-4516, which has been classified among non-canonical or rRNA-related small RNAs [59]. Such molecules are increasingly associated with ribosomal and nucleolar stress, conditions commonly observed in chronic inflammatory states [59,60]. In this context, the increased EV-associated abundance of miR-4516 may reflect persistent alterations in translational homeostasis, rather than acute regulatory responses.

Collectively, these findings indicate that EV-miRNAs may act as components of a coordinated network responding to chronic metabolic stress and other cellular stress-related processes. Within this framework, miR-4516 represents the most robust component of this coordinated response, while additional miRNAs contribute to a broader regulatory landscape reflecting immune, metabolic, and stress-adaptive processes.

From a translational perspective, the detection of EV-associated miRNAs in urine represents a non-invasive approach to monitor early biological alterations associated with metabolic stress in pediatric populations. Although the clinical applicability of these markers requires further validation, their ability to capture integrated stress responses highlights their potential value for early risk stratification and preventive strategies.

### Limitations

This study has several limitations that should be considered when interpreting the findings. First, the relatively small sample size may have reduced statistical power and limited the identification of additional AGE-associated miRNAs beyond hsa-miR-4516. Therefore, the results should be considered exploratory and require validation in larger, independent pediatric cohorts. Second, urinary AGEs were assessed using a spectrofluorimetric approach that quantifies total fluorescent AGEs. While this method is rapid and suitable for epidemiological studies, it does not distinguish specific AGE species or detect non-fluorescent compounds. Consequently, the observed associations reflect the overall AGE burden rather than individual AGE components. Third, the cross-sectional design precludes causal inference, and it remains unclear whether AGE accumulation drives changes in EV-associated miRNAs or whether both are influenced by shared metabolic or inflammatory factors. Another limitation of the present study is the lack of available biochemical markers of oxidative stress (e.g., TBARS and MDA). The inclusion of these parameters could have provided additional mechanistic insight into the metabolic pathways associated with AGE levels and EV-associated miRNA expression, as well as allowed further adjustment for potential metabolic confounders. Furthermore, only hsa-miR-4516 was validated by RT-qPCR. This miRNA was selected because it emerged as the most consistently observed candidate across the analytical approaches. Nevertheless, independent validation of additional differentially expressed miRNAs would further strengthen the reliability and generalizability of the sequencing findings and should be addressed in future studies. An additional limitation is that extracellular vesicle characterisation was not performed. Although RNA isolation was carried out using the exoRNeasy platform, which enriches for EV-associated RNA, the absence of orthogonal EV validation methods prevents definitive attribution of the detected miRNAs exclusively to bona fide extracellular vesicles. Therefore, the findings should be interpreted as deriving from EV-enriched urinary RNA preparations. In addition, the high inter-individual variability of EV cargo suggests that these signals should be interpreted as network-level responses rather than direct biomarkers. Finally, the functional mechanisms linking AGEs to EV-miRNA modulation were not experimentally investigated. In particular, the biological role of urinary EV-associated hsa-miR-4516 and its involvement in AGE-related inflammatory and cellular stress pathways remain to be clarified. Future longitudinal studies integrating targeted AGE profiling, biochemical characterisation, and functional miRNA analyses will be essential to define the mechanistic basis and clinical relevance of these findings.

## 5. Conclusions

This study provides novel evidence of an association between urinary AGEs and EV–associated miRNAs in children and adolescents. Among the identified candidates, hsa-miR-4516 emerged as the most consistent signal, supporting the concept of an EV-enriched miRNA profile associated with pediatric metabolic stress conditions. The urinary EV-miRNA profile reflects a restricted and coordinated response, suggesting that these markers may capture integrated biological adaptations associated with AGE-related inflammatory and cellular stress-response pathways rather than direct exposure levels. Despite the exploratory design and the relatively small sample size, these findings support a model in which EV-associated miRNAs act collectively as a network reflecting systemic stress responses. In this context, the non-invasive assessment of urinary EV-miRNAs represents a promising approach for the early detection of subclinical metabolic and inflammatory alterations in pediatric populations. Future longitudinal and mechanistic studies, integrating targeted AGE profiling and functional miRNA analyses in larger cohorts, are needed to validate the clinical relevance of these findings and clarify the role of EV-associated miRNAs in early metabolic risk stratification and precision prevention strategies.

## Figures and Tables

**Table 1:** Anthropometric and biochemical characteristics of the study participants.

	Sex			
Variable	Total (N = 94) ^1^	Males N = 51 ^1^	Females N = 43 ^1^	*p*-Value ^2^
Age (years)	11.60 (1.60)	11.66 (1.61)	11.52 (1.61)	0.663
BMI (z-score)	1.28 (0.99)	1.59 (1.03)	0.91 (0.80)	<0.001
hs-CRP	0.48 (1.05)	0.66 (1.32)	0.28 (0.55)	0.061
AGEs Levels (ln AGE)	5.63 (0.32)	5.64 (0.33)	5.62 (0.30)	0.764

^1^ Mean (SD). ^2^ Welch Two-Sample *t*-test.

**Table 2:** Top 30 differentially expressed EV-associated miRNAs according to AGE levels.

Name	Log2 Fold Change	*p*-Value	FDR *p*-Value
**hsa-miR-143-3p**	**−2.1061**	**0.0001**	**0.0205**
**hsa-miR-142-3p**	**−2.0038**	**0.0002**	**0.0205**
**hsa-miR-142-5p**	**−1.9778**	**0.0004**	**0.0247**
**hsa-miR-4516**	**1.2060**	**0.0003**	**0.0247**
**hsa-miR-483-5p**	**1.6103**	**0.0009**	**0.0485**
hsa-miR-10400-5p	−1.7747	0.0017	0.0728
hsa-miR-194-5p	1.5025	0.0020	0.0736
hsa-miR-223-3p	−1.7614	0.0030	0.0979
hsa-miR-223-5p	−1.4220	0.0089	0.2550
hsa-miR-126-3p	−0.7373	0.0097	0.2550
hsa-miR-10401-5p	1.0537	0.0179	0.3868
hsa-miR-3130-3p	0.7621	0.0191	0.3868
hsa-miR-185-5p	−0.5969	0.0188	0.3868
hsa-miR-186-5p	−0.6880	0.0358	0.4226
hsa-miR-191-5p	−0.3950	0.0296	0.4226
hsa-miR-10396b-5p	1.0083	0.0281	0.4226
hsa-miR-8071	1.3703	0.0294	0.4226
hsa-miR-222-5p	0.8741	0.0269	0.4226
hsa-miR-1275	0.5415	0.0326	0.4226
hsa-miR-429	−0.4926	0.0347	0.4226
hsa-miR-29b-3p	−0.5023	0.0318	0.4226
hsa-miR-139-5p	0.8991	0.0318	0.4226
hsa-miR-153-3p	1.2174	0.0370	0.4226
hsa-miR-16-5p	−0.3973	0.0436	0.4632
hsa-miR-486-5p	−0.5862	0.0440	0.4632
hsa-miR-744-5p	−0.4344	0.0509	0.5153
hsa-miR-204-3p	0.3732	0.0593	0.5778
hsa-miR-629-3p	0.8514	0.0634	0.5951
hsa-miR-181a-5p	−0.3841	0.0707	0.6194
hsa-miR-140-3p	−0.9357	0.0687	0.6194

**Table 3:** Literature-derived biological interpretation of FDR-significant EV-enriched urinary miRNAs (high-AGE versus low-AGE comparison).

miRNA	Direction in High-AGE Group	Possible Biological Significance	Potential Clinical/Pathophysiological Meaning	Supporting DOI Reference(s)
**hsa-miR-143-3p**	Downregulated Log2FC = −2.1061 *p* = 0.0001 FDR = 0.0205	Linked to cellular remodelling, proliferation, and inflammatory/fibrotic signalling, including PI3K/Akt, AKT/STAT3, and MAPK-related networks. Experimental evidence also supports a role in modulating tissue inflammation and fibrosis.	Reduced levels in the high-AGE group may suggest loss of a regulatory/protective axis involved in remodelling control, consistent with increased susceptibility to stress-driven inflammatory and fibrotic responses.	miR-143-3p and signalling networks: [46] miR-143 and inflammation/fibrosis: [47] AGE-related oxidative/inflammatory mechanisms: [8]
**hsa-miR-142-3p**	Downregulated Log2FC = −2.0038 *p* = 0.0002 FDR = 0.0205	Associated with immune regulation, particularly in macrophages and dendritic cells, where it modulates antigen uptake, processing, and presentation; also linked to IL-1β-dependent inflammatory signalling.	Lower EV levels may reflect immune-inflammatory dysregulation associated with AGE-related stress and support the presence of an altered immunomodulatory EV signature.	Antigen processing/presentation: [48] IL-1β-dependent neuroinflammation: [49]
**hsa-miR-142-5p**	Downregulated Log2FC = −1.9778 *p* = 0.0004 FDR = 0.0247	Implicated in macrophage polarisation and profibrogenic programs, particularly through IL-4/IL-13-driven pathways involving STAT6/SOCS1, which are relevant to chronic inflammation and fibrosis.	Downregulation may indicate perturbation of macrophage-associated regulatory pathways in the high-AGE group and is consistent with an EV profile linked to immune remodelling and fibrotic susceptibility.	Macrophage profibrogenic program: [50]
**hsa-miR-4516**	Upregulated Log2FC = 1.2060 *p* = 0.0003 FDR = 0.0247	Associated with oxidative stress control, mitochondrial function, and fibrosis-related pathways. Experimental studies suggest that restoring miR-4516 may reduce ROS accumulation, mitochondrial dysfunction, and fibrotic responses, including in EV-mediated contexts.	Increased levels may represent a compensatory/adaptive response to AGE-associated redox imbalance and tissue stress, making miR-4516 a potentially informative marker of EV-mediated stress adaptation.	Renal fibrosis/ROS/mitochondria: [51] EV-derived miR-4516 and lung fibrosis: [52]
**hsa-miR-483-5p**	Upregulated Log2FC = 1.6103 *p* = 0.0009 FDR = 0.0485	Linked to metabolic dysfunction, diabetes-related traits, fatty liver disease, nephropathy, and cell viability pathways. Circulating levels have been associated with cardiometabolic risk factors.	Upregulation is consistent with a link between higher AGE burden and metabolic stress-related EV signalling, suggesting possible relevance as a marker of cardiometabolic and glycoxidative stress adaptation.	miR-483 in metabolic/cardiometabolic disease: [53] miR-483-5p, steatosis/fibrosis/proliferation: [54]

**Table 4:** Top 30 differentially expressed EV-associated miRNAs identified through global transcriptional analysis based on generalised linear models (GLMs), unadjusted for covariates.

Name	Log2 Fold Change	*p*-Value	FDR *p*-Value
**hsa-miR-4516**	**0.6170**	**0.0004**	**0.0467**
**hsa-miR-122-5p**	**−1.0404**	**0.0007**	**0.0467**
**hsa-miR-7-5p**	**−0.6369**	**0.0011**	**0.0499**
hsa-miR-126-3p	−0.4659	0.0025	0.0849
hsa-miR-130a-3p	0.2679	0.0049	0.1313
hsa-miR-4492	0.4513	0.0069	0.1561
hsa-miR-3135b	−0.3527	0.0099	0.1904
hsa-miR-193b-5p	−0.3192	0.0133	0.2243
hsa-miR-206	0.6180	0.0186	0.2790
hsa-miR-30a-5p	0.1288	0.0236	0.3191
hsa-miR-151a-3p	−0.1167	0.0409	0.4419
hsa-miR-877-5p	−0.2693	0.0415	0.4419
hsa-miR-574-5p	−0.1372	0.0425	0.4419
hsa-miR-149-5p	−0.2133	0.0483	0.4657
hsa-miR-26a-5p	0.1010	0.0607	0.5464
hsa-miR-629-3p	0.5299	0.0804	0.5608
hsa-miR-30c-5p	0.2408	0.0809	0.5608
hsa-miR-429	−0.2048	0.0829	0.5608
hsa-miR-223-3p	0.6798	0.0841	0.5608
hsa-let-7f-5p	−0.0808	0.0857	0.5608
hsa-miR-7704	−0.1974	0.0872	0.5608
hsa-miR-3196	−0.2543	0.0918	0.5631
hsa-miR-9901	−0.3963	0.1034	0.6008
hsa-miR-30d-5p	0.0726	0.1107	0.6008
hsa-miR-486-3p	−0.1750	0.1138	0.6008
hsa-miR-375-3p	−0.3661	0.1194	0.6008
hsa-let-7b-5p	−0.0562	0.1245	0.6008
hsa-miR-12136	−0.2330	0.1303	0.6008
hsa-miR-204-3p	−0.1474	0.1347	0.6008
hsa-miR-23a-3p	0.307	0.1362	0.6008
hsa-miR-20a-5p	0.182	0.1414	0.6008

## Data Availability

Raw count matrices used in this manuscript are available from the corresponding author upon reasonable request.

## Supplementary Materials

The following supporting information can be downloaded at: https://www.mdpi.com/article/10.3390/antiox15091150/s1, Figure S1: Confirmatory RT-qPCR of miRNA in selected groups. Figure S2: Network representation of hsa-miR-4516 molecular targets. Figure S3: Tissue-specific expression of hsa-miR-4516 across datasets. Table S1: Top 30 differentially expressed EV-associated miRNAs identified through global transcriptional analysis based on generalised linear models adjusted for age, sex, and BMI z-score. Table S2: Top 30 differentially expressed EV-associated miRNAs identified through global transcriptional analysis based on generalised linear models adjusted for age, sex, BMI z-score, and hs-CRP. Table S3: List of miR-4516 molecular targets including experimentally validated interactions and high-confidence predicted targets, annotated with their biological functions and evidence levels.
