# Supplementary material for: Urinary Extracellular Vesicle-Derived miRNAs Reveal a Coordinated Stress-Response Network Linked to Advanced Glycation End-Products in Children and Adolescents
Source: Antioxidants (Basel). 2026 Sep 10;15(9):1150. doi: 10.3390/antiox15091150 (PMC13603886; doi:10.3390/antiox15091150)
Supplement: Supplementary file 1 [file antioxidants-15-01150-s001.zip › antioxidants-4497018-supplementary.pdf]

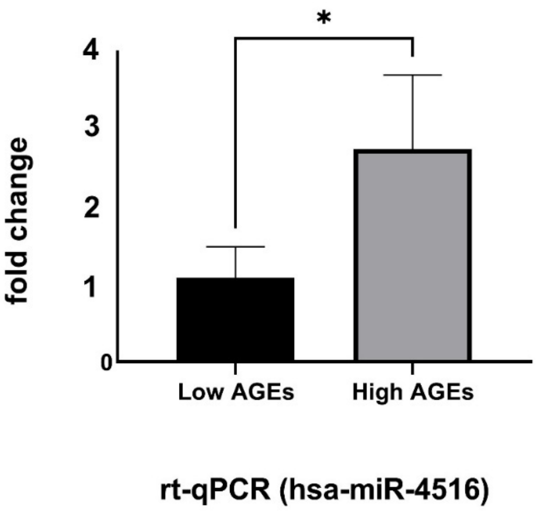

Supplementary Figure S1. Confirmatory RT-qPCR of miRNA in selected groups. Confirmatory real-time quantitative RT-PCR (RT-qPCR) in low AGE (n=47) and high AGE (n=47) groups was carried out using SYBR Green technology; samples were analysed in triplicate. Relative levels were calculated using DataAssist v3.1 software (Life Technologies, Thermo Fisher Scientific, Italy). Data are reported as mean ± SD. Statistical significance was assessed using a two-tailed Student's *t*-test. * *p* value = 0.0001.

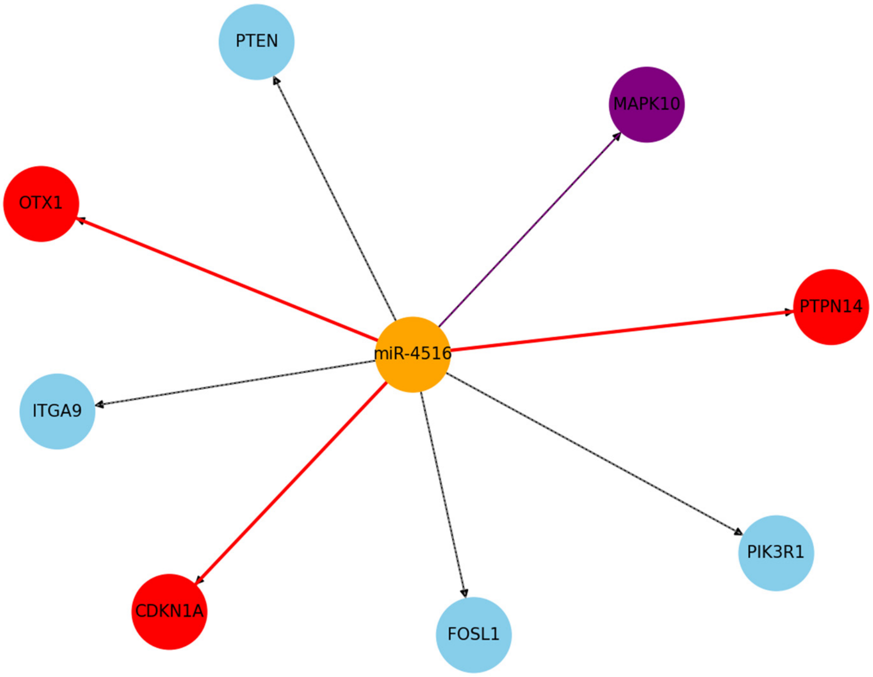

Supplementary Figure S2. Network representation of hsa-miR-4516 molecular targets. Direct targets (red nodes and solid edges) include PTPN14, CDKN1A, and OTX1, supported by experimental validation. Likely direct targets (purple) are supported by predictive and functional evidence. Indirect targets (blue) represent downstream effectors within signalling pathways modulated by hsa-miR-4516, including components of PI3K-AKT signalling (PTEN, PIK3R1), transcriptional regulation (FOSL1), and cell adhesion (ITGA9).

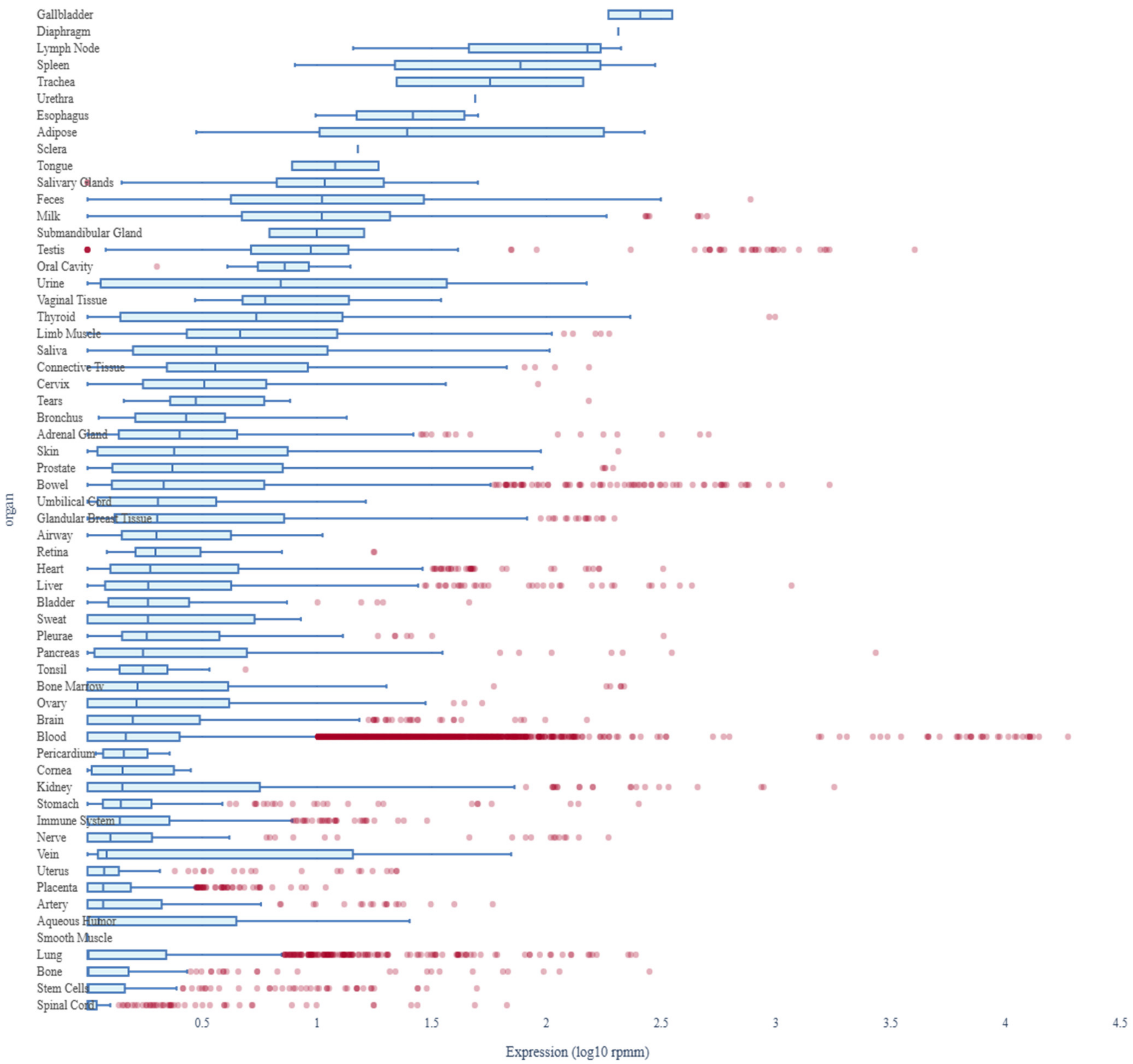

Supplementary Figure S3. Tissue-specific expression profile across datasets. The tissue-specific distribution of hsa-miR-4516 was systematically evaluated using the integrated analytical framework of the miRNATissueAtlas platform.

Supplementary Table S1. Top 30 differentially expressed EV-associated miRNAs identified through global transcriptional analysis based on generalised linear models adjusted for age, sex, and BMI z-score.

.Name	Log2 fold change	*p*-value	FDR *p*-value
hsa-let-7g-5p	-0.1214	0.0011	0.9683
hsa-miR-429	-0.3368	0.0050	1.0000
hsa-miR-671-5p	0.4387	0.0072	1.0000
hsa-miR-10401-5p	0.8428	0.0072	1.0000
hsa-miR-30e-5p	-0.1313	0.0120	1.0000
hsa-let-7f-5p	-0.1163	0.0142	1.0000
hsa-miR-4516	0.4370	0.0163	1.0000
hsa-miR-98-5p	-0.1213	0.0179	1.0000
hsa-miR-29b-3p	-0.2915	0.0187	1.0000
hsa-miR-100-5p	-0.2602	0.0212	1.0000
hsa-miR-141-3p	-0.1872	0.0230	1.0000
hsa-miR-3130-3p	0.4142	0.0241	1.0000
hsa-miR-26b-5p	-0.1055	0.0246	1.0000
hsa-miR-30e-3p	-0.1109	0.0275	1.0000
hsa-miR-206	0.5345	0.0380	1.0000
hsa-miR-122-5p	0.6218	0.0467	1.0000
hsa-miR-421	0.2928	0.0494	1.0000
hsa-miR-103a-3p	-0.1343	0.0509	1.0000
hsa-miR-16-5p	-0.1650	0.0533	1.0000
hsa-miR-32-3p	-0.3280	0.0570	1.0000
hsa-miR-3173-3p	-0.4045	0.0749	1.0000
hsa-miR-27b-5p	0.2146	0.0757	1.0000
hsa-miR-126-3p	-0.2758	0.0761	1.0000
hsa-miR-1268a	0.1575	0.0779	1.0000
hsa-miR-1-3p	-0.3580	0.0884	1.0000
hsa-miR-125a-3p	-0.1639	0.0918	1.0000
hsa-miR-184	0.4259	0.0918	1.0000
hsa-miR-362-5p	-0.1558	0.1033	1.0000
hsa-miR-9-5p	-0.4545	0.1110	1.0000
hsa-miR-27b-3p	0.3162	0.1169	1.0000
hsa-miR-4508	0.2188	0.1214	1.0000

Full results including non-FDR-significant associations are provided for completeness.

Supplementary Table S2. Top 30 differentially expressed EV-associated miRNAs identified through global transcriptional analysis based on generalised linear models adjusted for age, sex, BMI z-score, and hs-CRP.

Name	Log2 fold change	*p*-value	FDR *p*-value
hsa-let-7g-5p	-0.1224	0.0014	0.8762
hsa-miR-100-5p	-0.3470	0.0020	0.8762
hsa-miR-4516	0.4991	0.0060	1.0000
hsa-miR-429	-0.3272	0.0085	1.0000
hsa-let-7f-5p	-0.1237	0.0107	1.0000
hsa-miR-10401-5p	0.8277	0.0112	1.0000
hsa-miR-30e-5p	-0.1356	0.0117	1.0000
hsa-miR-206	0.6587	0.0120	1.0000
hsa-miR-671-5p	0.4198	0.0127	1.0000
hsa-miR-98-5p	-0.1264	0.0164	1.0000
hsa-miR-26b-5p	-0.1103	0.0222	1.0000
hsa-miR-3130-3p	0.4282	0.0239	1.0000
hsa-miR-29b-3p	-0.2808	0.0288	1.0000
hsa-miR-141-3p	-0.1857	0.0289	1.0000
hsa-miR-16-5p	-0.1861	0.0329	1.0000
hsa-miR-103a-3p	-0.1451	0.0407	1.0000
hsa-miR-30e-3p	-0.1056	0.0415	1.0000
hsa-miR-421	0.3078	0.0477	1.0000
hsa-miR-184	0.4860	0.0552	1.0000
hsa-miR-1-3p	-0.4103	0.0557	1.0000
hsa-miR-126-3p	-0.3002	0.0600	1.0000
hsa-miR-3173-3p	-0.4122	0.0791	1.0000
hsa-miR-32-3p	-0.3129	0.0824	1.0000
hsa-miR-126-5p	-0.5044	0.0827	1.0000
hsa-miR-122-5p	0.5384	0.0922	1.0000
hsa-miR-125a-3p	-0.1629	0.1079	1.0000
hsa-miR-1268a	0.1421	0.1189	1.0000
hsa-miR-27b-3p	0.3215	0.1235	1.0000
hsa-miR-200a-3p	-0.1380	0.1274	1.0000
hsa-miR-23a-3p	0.3185	0.1278	1.0000
hsa-miR-4508	0.2151	0.1372	1.0000

Full results including non-FDR-significant associations are provided for completeness.

Supplementary Table S3. List of miR-4516 molecular targets including experimentally validated interactions and high-confidence predicted targets, annotated with their biological functions and evidence levels.

Gene	Evidence Level	Validation Method	Biological Context	Functional Role	Putative Relevance to AGE-Related Mechanisms
PTPN14	High	Luciferase + functional assays	Glioblastoma	Hippo pathway signalling and cell growth regulation	Potential mediator of proliferative responses associated with AGE-RAGE signalling
CDKN1A	High	Functional studies	Cancer cells	Cell-cycle arrest and stress-induced senescence	Regulates cellular stress responses and senescence linked to AGE accumulation
OTX1	High	Luciferase + expression studies	Pancreatic cancer	Transcription factor involved in proliferation, migration, and epithelial-mesenchymal transition	Potential regulator of tissue remodelling and stress-adaptive responses
MAPK10	Moderate	Predicted + functional evidence	Stress models	Stress-activated MAP kinase involved in apoptosis and oxidative-stress signalling	Potential mediator of ROS-dependent signalling pathways activated by AGEs
BTK	Moderate	Expression association	Colorectal cancer	Inflammation-related signaling	Possible contribution to inflammatory pathways associated with chronic AGE exposure
PTEN	Predicted	TargetScan + literature support	Multiple tissues	PI3K/AKT pathway regulation and cell survival	Functionally linked to oxidative stress and metabolic regulation
PIK3R1	Predicted	TargetScan/miRDB	General	Regulatory subunit of PI3K involved in metabolic and stress-response signalling.	Potential mediator of AGE-associated metabolic adaptations
PPP2R1A	Predicted	TargetScan	Signal transduction	Scaffold subunit of PP2A regulating multiple signalling pathways	May influence redox-sensitive signalling networks
FOSL1	Predicted	TargetScan + EV studies	Cancer/EV biology	AP-1 transcription factor involved in oxidative-stress responses and inflammation	Potential contributor to transcriptional programs activated by AGE-induced stress
ITGA9	Predicted	TargetScan	Cell adhesion	Cell adhesion and extracellular matrix interactions	May participate in tissue-remodelling processes associated with chronic metabolic stress
NFIX	Low	TargetScan	Development	Transcription factor	Indirect stress adaptation
RNF10	Low	Literature association	Protein turnover	Protein ubiquitination and transcriptional regulation	Potential involvement in cellular adaptation to proteotoxic stress
KHNYN	Low	TargetScan	RNA metabolism	RNA metabolism and antiviral response	Possible indirect role in cellular stress-response pathways
GRAMD2	Low	TargetScan	Lipid transport	Membrane-associated protein involved in lipid transport and organelle contact sites	Potential role in membrane homeostasis under metabolic stress
SPOCK1	Low	TargetScan	Extracellular matrix	Extracellular matrix organisation and tissue remodelling	Potential contributor to structural remodelling associated with chronic AGE exposure

Evidence levels were assigned according to the strength of available target-validation data. High: direct experimental validation of miRNA-target interaction by luciferase reporter assays and/or functional studies demonstrating target regulation. Moderate: target supported by expression-based evidence and functional association studies. Low: target supported by limited experimental evidence or indirect literature reports. Predicted: target identified exclusively through curated target-prediction databases without direct experimental validation. Functional annotations and putative AGE-related relevance should be interpreted as plausible mechanistic links rather than confirmed AGE-specific effects.
